# Mitochondrial Transfer Improves Cardiomyocyte Bioenergetics and Viability in Male Rats Exposed to Pregestational Diabetes

**DOI:** 10.3390/ijms22052382

**Published:** 2021-02-27

**Authors:** Eli J. Louwagie, Tricia D. Larsen, Angela L. Wachal, Tyler C.T. Gandy, Michelle L. Baack

**Affiliations:** 1 Sanford School of Medicine, University of South Dakota, Sioux Falls, SD 57105, USA; 2 Environmental Influences on Health and Disease Group, Sanford Research, Sioux Falls, SD 57104, USA; Tricia.Larsen@SanfordHealth.org (T.D.L.); angela.wachal@k12.sd.us (A.L.W.); tyler.gandy@sanfordhealth.org (T.C.T.G.); 3 Boekelheide Neonatal Intensive Care Unit, Sanford Children’s Hospital, Sioux Falls, SD 57117, USA

**Keywords:** mitochondria, diabetic pregnancy, developmentally programmed heart disease, mitochondrial transfer

## Abstract

Offspring born to diabetic or obese mothers have a higher lifetime risk of heart disease. Previously, we found that rat offspring exposed to late-gestational diabetes mellitus (LGDM) and maternal high-fat (HF) diet develop mitochondrial dysfunction, impaired cardiomyocyte bioenergetics, and cardiac dysfunction at birth and again during aging. Here, we compared echocardiography, cardiomyocyte bioenergetics, oxidative damage, and mitochondria-mediated cell death among control, pregestational diabetes mellitus (PGDM)-exposed, HF-diet-exposed, and combination-exposed newborn offspring. We hypothesized that PGDM exposure, similar to LGDM, causes mitochondrial dysfunction to play a central, pathogenic role in neonatal cardiomyopathy. We found that PGDM-exposed offspring, similar to LGDM-exposed offspring, have cardiac dysfunction at birth, but their isolated cardiomyocytes have seemingly less bioenergetics impairment. This finding was due to confounding by impaired viability related to poorer ATP generation, more lipid peroxidation, and faster apoptosis under metabolic stress. To mechanistically isolate and test the role of mitochondria, we transferred mitochondria from normal rat myocardium to control and exposed neonatal rat cardiomyocytes. As expected, transfer provides a respiratory boost to cardiomyocytes from all groups. They also reduce apoptosis in PGDM-exposed males, but not in females. Findings highlight sex-specific differences in mitochondria-mediated mechanisms of developmentally programmed heart disease and underscore potential caveats of therapeutic mitochondrial transfer.

## 1. Introduction

Diabetes and obesity during pregnancy expose the fetus to excess circulating glucose and lipids, inciting fetal hyperinsulinemia and fuel-mediated heart disease at birth [1,2,3,4] and later in life [5,6,7], yet prevention is hindered because mechanisms are not well understood. Work from our lab [8,9,10,11,12] and others [13,14,15] suggests that mitochondria play a central, pathogenic role, specifically in offspring exposed to late-gestation diabetes mellitus (LGDM) and maternal high-fat (HF) diet. This study aimed to answer key remaining questions. Specifically, (1) does earlier and longer exposure associated with pregestational diabetes (PGDM) cause more severe mitochondrial dysfunction? (2) Are mitochondria primary mediators of cardiac disease, regardless of fuel transport, storage, or enzymatic flux?

It is well known that mitochondria are key regulators of cardiac development, function, and disease [16]. Their most well-known role, energy production, is critical to highly metabolic organs like the heart. Moreover, mitochondria mediate cell fate including proliferation, differentiation, and viability that could alter normal development [16]. Using a rat model of LGDM and HF diet that prenatally exposes offspring to maternal hyperglycemia, hyperlipidemia, and fetal hyperinsulinemia in the last third of pregnancy, we found that newborn rats exposed to diabetic pregnancy have larger hearts with diastolic and systolic dysfunction [9,10] similar to humans [2]. Their isolated cardiomyocytes (CM) have intrinsic mitochondrial dysfunction with impaired dynamics, bioenergetics, and sex-specific differences in mitochondrial complex function that is worsened in combination with maternal HF diet [8,9,10]. As adults, LGDM-exposed male offspring have faster mitochondrial membrane potential (MMP) loss and mitochondria-mediated death under stress [9]. Others have described lasting cardiovascular consequences resulting from metabolic memory, the concept that exposure to even transient hyperglycemia imparts lasting effects through epigenetic modification and oxidative stress [17,18]. Yet, our studies suggest that beyond transient hyperglycemia, developmentally programmed heart disease is more complex as lipids and insulin, rather than just hyperglycemia, appear to play important roles. Indeed, our own LGDM and HF diet model demonstrate significant diet-mediated changes in the myocardial epigenome and transcriptome [8,11], particularly alongside lipid-associated oxidative stress [10]. Given our previous findings, we hypothesized that maternal PGDM alongside a maternal HF diet would expose the developing offspring to hyperglycemia during ovulation, fertilization, implantation, and organogenesis to cause even worse mitochondrial dysfunction in developing CM (compared to LGDM exposure) potentially contributing to cardiac birth defects as well as neonatal cardiomyopathy by higher rates of mitochondria-mediated cell death.

To mechanistically isolate and test the role of mitochondria in CM bioenergetic dysfunction and cell fate, we combined mitochondrial transfer (often termed “mitochondrial transplantation” in in vivo models) with Seahorse extracellular flux analyses, confocal imaging for mitophagy, and our carbonyl cyanide-4-phenylhydrazone (FCCP) Challenge, which quantifies MMP loss and cell death responses to respiratory stress, much like a “heart attack in a dish” [9]. Mounting in vitro and in vivo studies have shown that isolated mitochondria with intact respiration can be injected or perfused into the heart, where they are quickly internalized by CM [19,20,21] via actin-dependent endocytosis [22]. Once inside host cells, donor mitochondria escape from endosomes and fuse with the host mitochondrial network [23]. This incorporation of healthy donor mitochondria leads to improved cellular and cardiac function by increasing myocardial respiration and ATP production [20,24], upregulating expression of cardioprotective cytokines and mitochondrial proteins involved in respiration [24], and by replacing damaged mitochondrial (mt)DNA [22] to ultimately limit ischemic damage and improve cardiac function [25,26]. In clinical trials, mitochondrial transplantations offer an emerging therapeutic tool in the treatment of cardiomyopathy following ischemia-reperfusion injury (IRI) [26,27,28]. Here, we apply this technique to understand the role of mitochondria in developmentally programmed heart disease. Findings uncover sex-specific mitochondria-mediated mechanisms of developmentally programmed heart disease and therapeutic responses to mitochondrial transfer.

## 2. Results

### 2.1. PGDM and Maternal HF Diet Incite Maternal Glucolipotoxicity and Increase Perinatal Mortality

This study evaluated the effects of PGDM using 398 offspring of 30 litters (10 control, nine PGDM-exposed, six HF diet-exposed, and five combination-exposed). Maternal and newborn characteristics are shown in Table 1. As expected, PGDM incites maternal hyperglycemia before and throughout pregnancy. Maternal HF diet tends to worsen diabetes by raising maternal hyperglycemia (349 ± 21 vs. 413 ± 27mg/dL, *P* = 0.086 by t-test), insulin needs (85 ± 13 vs. 115 ± 14units throughout pregnancy, *P* = 0.163), and maternal ketones (0.73 ± 0.06 vs. 2.40 ± 0.29mmol/L, *P* < 0.0001), which can readily cross the placenta as an additional exposure for developing offspring. Maternal HF diet does not significantly affect maternal weight, but it increases circulating triglycerides, especially in diabetic dams. As in our LGDM model, maternal HF diet increases perinatal mortality seven- to eightfold despite no change in litter size (implantations). Unlike LGDM, PGDM-exposed offspring have greater birthweights and lower blood glucose levels than control offspring, reflecting more profound fetal hyperinsulinemia seen here and in human infants of diabetic mothers [29].

### 2.2. Offspring Exposed to PGDM and HF Diet Manifest Cardiac Dysfunction

**Figure 1:**
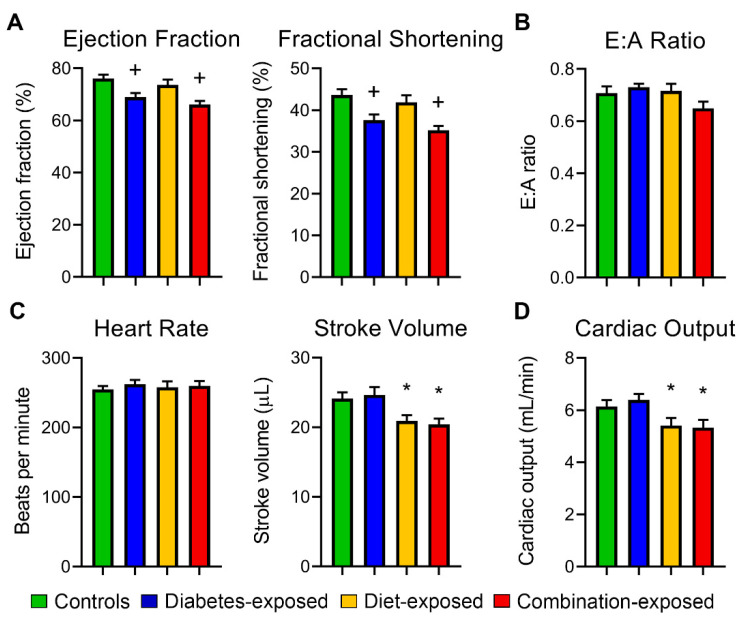
Diabetes- and high-fat (HF) diet-exposed newborn males had significant cardiac dysfunction at birth. (**A**) Systolic function, (**B**) diastolic function, (**C**) heart rate and stroke volume were determined in P1 offspring by echocardiography. (**D**) Cardiac output was calculated from (**C**). *N* = 12–19 offspring/group. Significant differences (*p* ≤ 0.05): ^+^ diabetes or * diet effect by 2-way NOVA.

Neonatal cardiac structure and function from cohort 1 were evaluated by morphometric measures and echocardiography (Figure 1; Table S1. Male offspring exposed to maternal diabetes had 4% greater heart:body weight ratios (Table S1) and impaired systolic function per 7.3% lower ejection fraction and 6.4% lower fractional shortening (Figure 1A). Unlike in our LGDM model [2,9], diastolic function per E:A ratio was not different between groups (Figure 1B). Stroke volume was 14% lower in HF diet-exposed offspring leading to significantly lower cardiac output (Figure 1C,D). As with LGDM [9,10], combination-exposed offspring had the poorest systolic and diastolic function and cardiac output. Pulmonary hypertension was not found in PGDM- or HF diet-exposed offspring (Table S1).

### 2.3. Mitochondria Isolated from Newborn Rat Hearts Retain Respiratory Function, Are Highly Concentrated, and Are Relatively Free of Nuclear Contamination

Approximately 8–9 newborn hearts were pooled for each mitochondrial isolation, yielding 1620 ± 110 µg mitochondria. To demonstrate respiratory competence of donor mitochondria, ATP levels were quantified with or without ADP added. Indeed, isolated mitochondria retain respiratory capacity following isolation (Figure S1A). Protein assays and immunoblotting demonstrated high concentration and good purity of mitochondrial isolates per relative expression levels of mitochondrial proteins cyclophilin D (CYPD) and voltage-dependent anion channel (VDAC, or porin), nuclear protein lamin A, and cytoskeletal proteins β-actin and β-tubulin (Figure S1B). Compared to whole-heart homogenate, CYPD and VDAC are more highly concentrated in mitochondrial isolates, indicating concentrated myocardial mitochondria. Lamin A is undetectable, indicating minimal nuclear contamination. Low levels of β-actin and β-tubulin are present in mitochondrial isolates, but this may support retained mitochondrial function as mitochondria associate with these proteins for vital cell functions [30,31].

### 2.4. Donor Mitochondria Are Increasingly Internalized by CM Over Time

**Figure 2:**
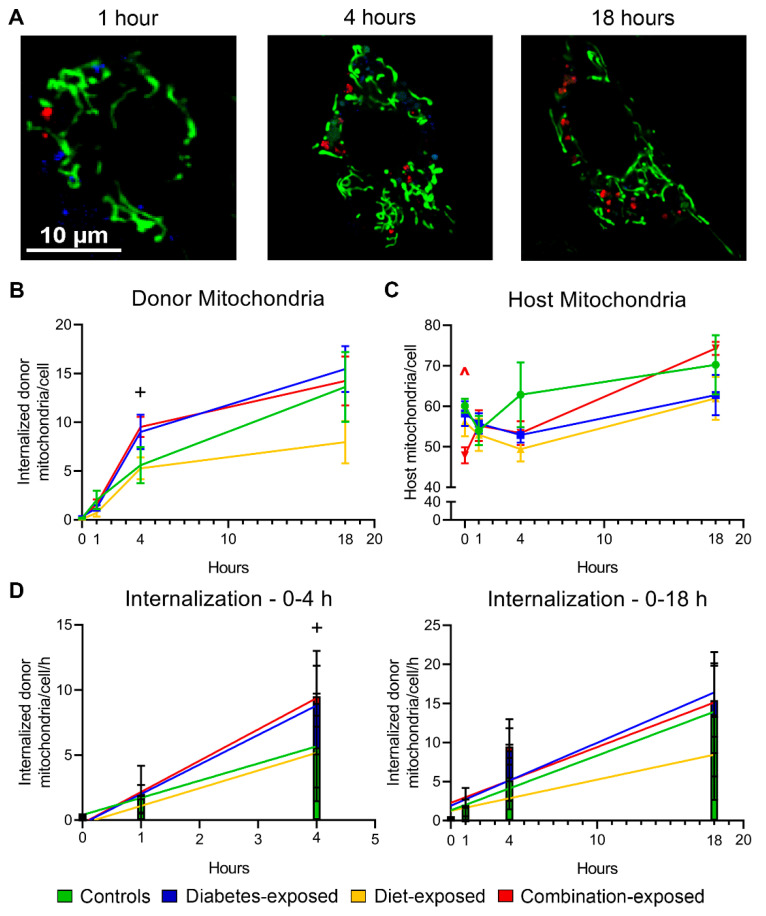
Donor mitochondria are internalized by cardiomyocytes at faster rates with diabetes exposure. (**A**) Representative confocal live-cell images of MitoTracker Green- and LysoTracker Blue-stained cardiomyocytes co-incubated with pHrodo Red-stained donor mitochondria. Numbers of internalized donor mitochondria (**B**) and host mitochondria (**C**) over time. (**D**) Rates of donor mitochondria internalization in the first four hours of co-incubation (left) and across 18 h (right). Data represent mean ± SEM. N = 5–6 males/group. Significant differences (*p* ≤ 0.05): ^+^ diabetes effect by 2-way ANOVA, ^ group effect by 1-way ANOVA and Dunnett post hoc test when interaction was present by 2-way ANOVA.

Optimal dosing for mitochondrial transfer was determined using dose-response assays for each well/plate and timing of experiments. This was determined to be 30 µg of donor mitochondria for 80,000 CM in 24 well Seahorse XFe plates (mitochondrial stress test [MST] and glycolytic stress test [GST]) and 60 µg mitochondria for 150,000 CM per 35 mm FluoroDish (confocal studies) (Figure S2). Next, CM were stained with MitoTracker Green (for host mitochondria) and LysoTracker Blue (for lysosomes), imaged for baseline characteristics, and then co-incubated with isolated donor mitochondria labeled with pHrodo Red. Baseline size and shape of host CM and mitochondria were similar between groups (Figure S3A-C, E) with the exception of diet-exposed CM having approximately 20% smaller mitochondria by cross-sectional area (Figure S3D). Imaging after 1, 4, and 18 h co-incubation then showed that control and exposed CM increasingly internalize and retain donor mitochondria (Figure 2A,B). Combination-exposed CM have fewer mitochondria at baseline, but after co-incubation with donor mitochondria the number rises (Figure 2C) leading to 50% more mitochondria by 18 h. This apparent increase in mitochondria/cell may be due to MitoTracker Green, which labeled host mitochondria leaching to internalized donor mitochondria or transfer-stimulated mitochondrial biogenesis. To assure cellular results were truly due to mitochondrial transfer rather than dye leaching, additional validation of internalization using non-dye studies was completed as described in *Methods* Section 4.4. Additionally, transfer-stimulated mitochondrial biogenesis is supported by the average number of total mitochondria within the cell at 18 h being higher than the sum of baseline host mitochondria and donor mitochondria at 1 h (Figure 2B,C). Linear regression analysis demonstrates that PGDM-exposed CM have faster uptake of donor mitochondria in the first four hours of co-incubation (Figure 2D) resulting in approximately double the number of donor mitochondria per cell at 4 h compared to control or HF-diet exposed CM (Figure 2B). Control CM take up donor mitochondria more slowly in the first 4 h but “catch up” by 18 h so that the overall numbers of internalized mitochondria are similar.

### 2.5. Internalized Donor Mitochondria Fuse with Host Mitochondria and Stimulate Mitophagy

Rates of colocalization were quantified as markers of mitochondrial fusion (donor with host mitochondria) and mitophagy (mitochondria with lysosomes) as shown in Figure S4A. In all CM, fusion of host and donor mitochondria is highest after one hour of internalization and decreases thereafter (Figure S4B). Number of lysosomes/cell at baseline is not different among groups (Figure S4C). After transfer, the number of host lysosomes increases significantly from baseline to 18 h in control (*P* = 0.031) and combination-exposed CM (*P* = 0.020) (Figure S4C). In all groups, mitophagy of donor mitochondria increases over time (*P* < 0.060), while mitophagy of host mitochondria decreases (Figure S4D); this could reflect turnover of donor mitochondria contents that improve the “quality” of host mitochondria. PGDM-exposed CM have a higher level of mitophagy of donor mitochondria than controls after 4 h co-incubation (*P* = 0.033).

### 2.6. Diabetes and Diet-Exposed Male Cardiomyocytes Have Greater Glycolytic Capacities 

Because diabetes impairs glycolysis in humans [32] and LGDM-exposed offspring [9], we used a GST to evaluate the effects of mitochondrial transfer in our PGDM model. In contrast to LGDM-exposed CM [9,10], maximal and reserve glycolytic capacities are higher in PGDM-exposed and diet-exposed male CM in cohort 1 (Figure S5A). PER was calculated to differentiate lactate-derived acidification (anaerobic glycolysis) from CO_2_-derived acidification (aerobic respiration). This confirmed that higher glycolytic capacities in PGDM-exposed males are due to higher anaerobic glycolysis (Figure S5B). Although the trend was similar, statistical significance was not reached in cohort 2 (Figure S5C,D). Other than a small decline in baseline anaerobic glycolysis in PGDM-exposed females, mitochondrial transfer did not change glycolytic capacities (Figure S5D). 

### 2.7. Diabetes-Exposed Male, But Not Female, CM Consume More Oxygen for ATP Production

**Figure 3:**
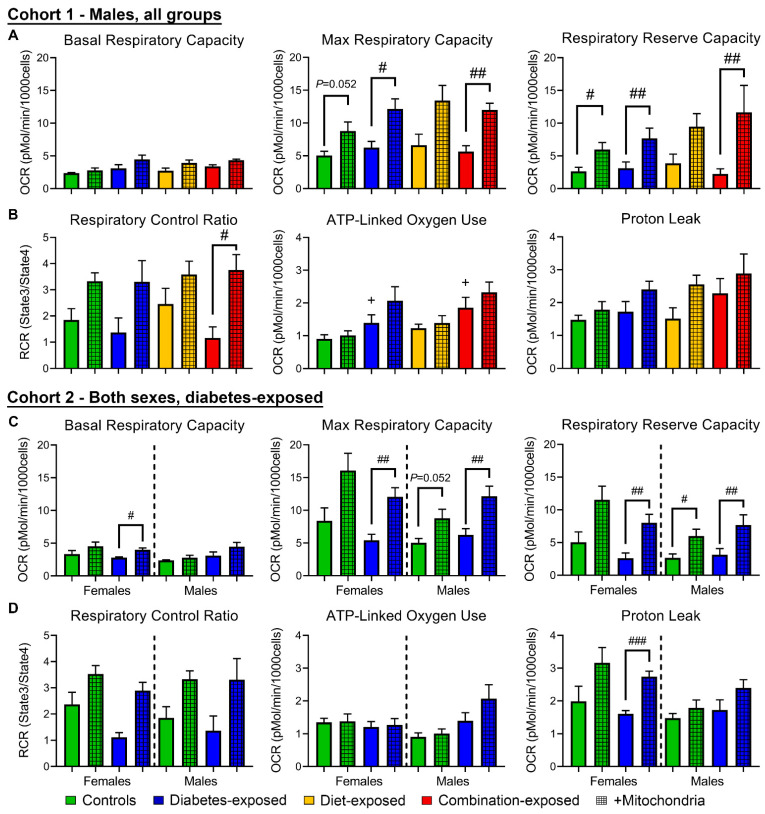
PGDM-exposed males, but not females, use more oxygen for ATP production, and while mitochondrial transfer may benefit males, it may put females at greater risk of ROS production. (**A**) Basal, FCCP-stimulated maximal, and reserve respiratory capacities and (**B**) respiratory control ratios, oxygen consumed in ATP production, and proton leak of male cardiomyocytes (CM) determined by mitochondrial stress test supplemented with mitochondrial transfer. (**C**,**D**) Respiratory parameters from PGDM-exposed CM of both sexes. *N* = 4–6 per group. Data represent mean ± SEM. *P* ≤ 0.05: ^+^ diabetes effect by 2-way ANOVA, ^#^ mitochondrial effect by 1-way ANOVA. ^##^ *P* < 0.01, ^###^ *P* < 0.001.

Unlike LGDM-exposed CM, which had significantly impaired respiration [9], basal, maximal, and reserve respiratory capacities, respiratory control ratio (RCR) and proton leak were similar among PGDM-exposed male CM (Figure 3A,B); however, they had higher ATP-linked oxygen consumption (Figure 3B). PGDM-exposed female CM were similar to controls including ATP-linked oxygen consumption (Figure 3C,D). This is in sharp contrast to previous findings from our LGDM-exposed model which demonstrated significant respiratory impairment in perinatal day (P)1 CM [8,9]. Because real-time data were normalized to the number of live cells at the end of the run (after FCCP) rather than the number plated, we worried that our data were confounded by cell death during the assay. 

### 2.8. PGDM-Exposed CM Have Inefficient ATP Production and Oxidative Damage at Baseline and Faster MMP Loss with More Cell Death under Metabolic Stress

**Figure 4:**
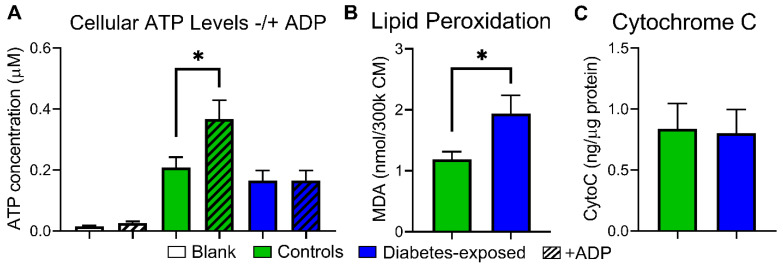
PGDM-exposed cardiomyocytes suffer from impaired phosphorylation and increased lipid peroxidation. (**A**) Cellular ATP levels with and without addition of ADP. Left two columns show media (negative control). (**B**) Malondialdehyde (MDA) was measured as a surrogate marker for lipid peroxidation. (**C**) Cytosolic cytochrome C levels at baseline, i.e., not under stress. *N* = 8–10 per group (sexes combined). Data represent mean ± SEM. * *P* ≤ 0.05 by 1-way ANOVA (**A**) or T-test (**B**).

ATP production, oxidative damage, and mitochondria-mediated responses to stress were investigated in PGDM-exposed offspring of both sexes (cohort 2). Although control and PGDM-exposed CM have similar baseline ATP content, control cells can readily phosphorylate ADP to ATP, while PGDM-exposed cells cannot (Figure 4A). Impaired phosphorylation of ADP despite higher oxygen consumed in ATP production (Figure 3B) suggests greater ROS production with downstream oxidative damage. Indeed, we found MDA levels were 60% higher in PGDM-exposed CM (Figure 4B), primarily due to males whose levels were nearly 80% higher than controls. Although cytochrome C levels were similar between groups (Figure 4C), authors suspected these cellular changes would cause faster cell death under metabolic stress with FCCP.

**Figure 5:**
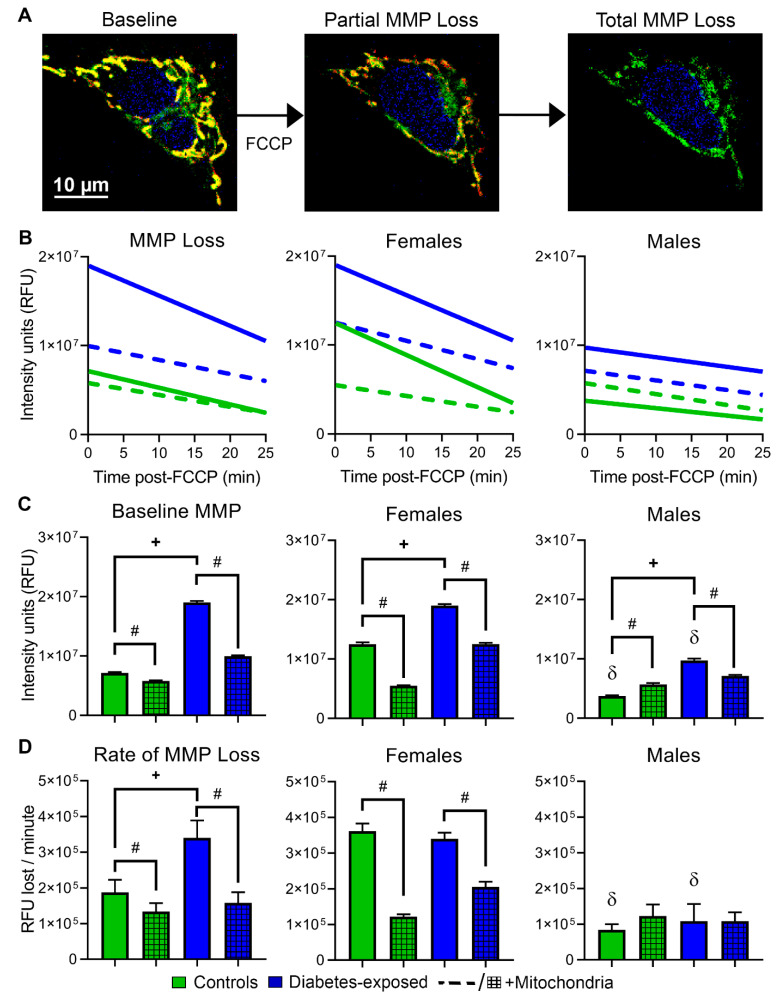
Females and PGDM-exposed cells have stronger mitochondrial membrane potential (MMP), but they lose it faster following metabolic stress. (**A**) Representative images of CM stained with MitoTracker Green, Hoechst, and MitoTracker Red. Once treated with FCCP, mitochondria lose their MMP and trigger cell death through intrinsic apoptosis. (**B**) MMP loss was analyzed before and following FCCP-induced stress by linear regression analysis. (**C**) Baseline MMP and (**D**) rate of MMP loss over 25 min of FCCP-induced stress. *N* = 4–5 per sex per group. Data represent mean ± SEM. *P* ≤ 0.05: ^+^ diabetes, ^#^ mitochondrial, or δ sex-specific effect (only lower sex marked) by 1-way ANOVA.

**Figure 6:**
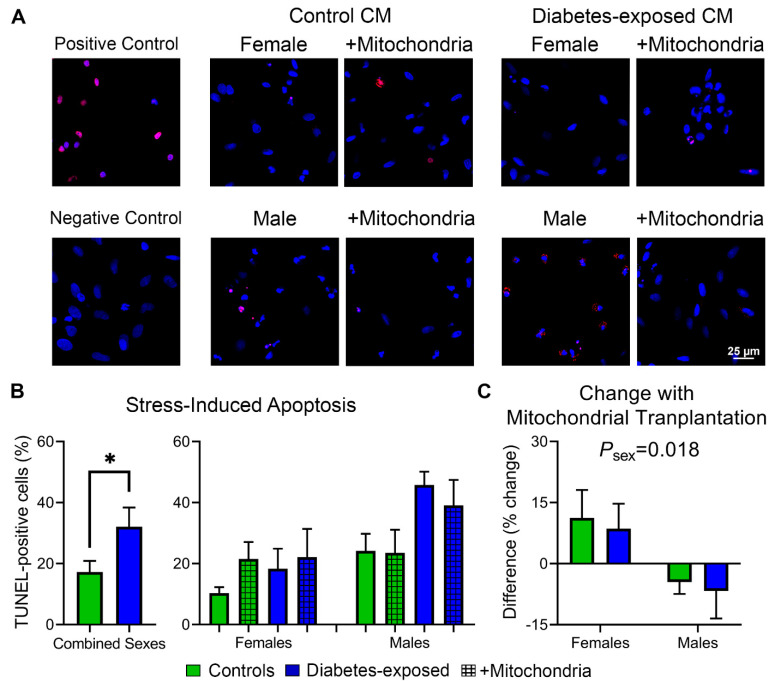
PGDM-exposed cardiomyocytes of both sexes are at greater risk of stress-induced apoptosis; mitochondrial transfer lowers this risk in males but worsens it in females. (**A**) Representative CM labeled with 4′,6-diamidino-2-phenylindole (DAPI) and transferase dUTP nick end labeling (TUNEL) for apoptotic cell death following FCCP Challenge. DNase treatment was used as a positive control. TUNEL reaction without enzyme was used as a negative control. (**B**) Percentage of TUNEL-positive cells following FCCP Challenge. Males have higher numbers of TUNEL-positive cells regardless of group (*P* = 0.005 by 1-way ANOVA; not demarcated). (**C**) Difference in TUNEL-positive cells with mitochondrial transfer. *N* = 4–5 per sex per group using 138 ± 10 cells per plate from 7–10 systematically imaged fields. Data represent mean ± SEM. * *P* ≤ 0.05 by 1-way ANOVA.

CM were subjected to FCCP Challenge [9] to measure rates of MMP loss (Figure 5) and apoptosis (Figure 6) under metabolic stress. Even before FCCP-induced stress, PGDM-exposed CM of both sexes have greater MMP (Figure 5C). Interestingly, females of both groups have higher MMP than males that may reflect superior mitochondrial function and their lesser respiratory boost from mitochondrial transfer [33]. Control females and PGDM-exposed CM of both sexes treated with mitochondrial transfer had lower MMP at baseline that affected rates of MMP loss after FCCP (Figure 5D); CM that started from higher levels fell faster, possibly a result of having “more to lose.” Next, it was imperative to determine whether these consequences increase stress-induced apoptosis as previously found in LGDM-exposed adult males [9]. Quantifying TUNEL-positive cells after FCCP Challenge demonstrates that PGDM-exposed CM suffer from significantly greater rates of stress-induced apoptosis than controls; this is highlighted by more than double the number of TUNEL-positive cells in this group (17 ± 7% vs. 41 ± 15%; Figure 6A,B). Males had more apoptotic CM than females regardless of exposure (*P* = 0.005).

### 2.9. Mitochondrial Transfer Improves Cellular Respiration and Reduces Stress-Induced Apoptosis in PGDM-Exposed Males but Increases It in Females

Mitochondrial transfer increases maximal and reserve capacities in all cohort 1 CMs, reaching significance in PGDM- and most evidently combination-exposed CM, whose RCR is also significantly higher post-transfer (Figure 3A,B). Cohort 2 demonstrated that like males, mitochondrial transfer significantly increases reserve capacity of PGDM-exposed female CMs (Figure 3C). However, PGDM-exposed females have also increased proton leak (Figure 3D) which may uncouple oxygen from ATP generation and increase MMP loss, ROS production [34], and the risk of apoptotic cell death (Figure 3D).

Effects of mitochondrial transfer were also sex-specific (*P* = 0.049) (Figure 6B). Transfer reduced the number of TUNEL-positive CM by 4.5 and 6.7% in control and PGDM-exposed males, respectively. However, transfer increased the number of TUNEL-positive cells by 11.2 and 8.6% in control and PGDM-exposed females, respectively (Figure 6C).

### 2.10. Intact mtDNA Plays a Major Role in Respiratory Boost of Mitochondrial Transfer

**Figure 7:**
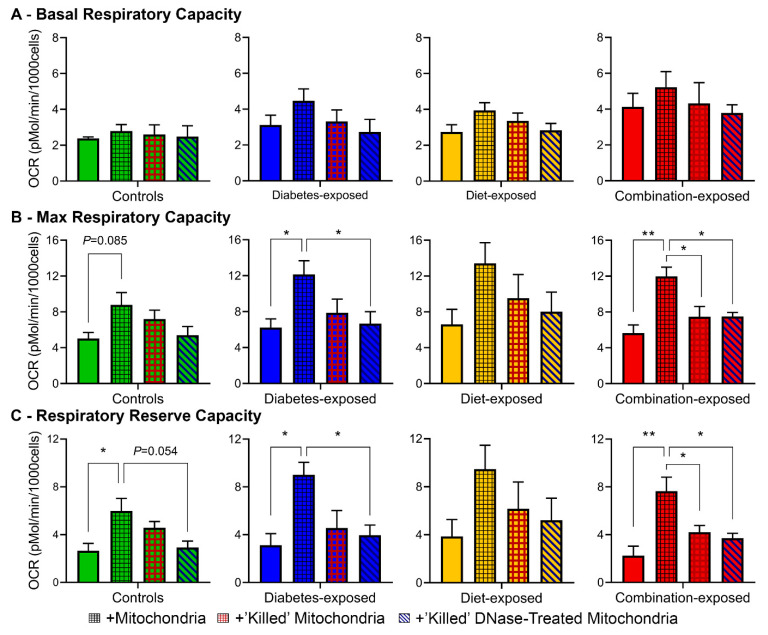
MtDNA plays a role in respiratory boost of mitochondrial transfer. (**A**) Basal, (**B**) FCCP-stimulated maximal, and (**C**) reserve respiratory capacities of CM treated with sham (solid), living–respiring (black hashing), “killed” (red hashing), and “killed”+DNase-treated mitochondria (diagonal). *N* = 4–6 per group. Data represent mean ± SEM. *P* ≤ 0.05: ^#^ mitochondrial effect by 1-way ANOVA. ** *P* < 0.01.

Two control treatments were used alongside mitochondrial transfer to delineate mechanisms of respiratory boost (Figure 7). The first used mitochondria “killed” by snap freezing to rupture membranes and heating to denature complex proteins; importantly, mtDNA may withstand both of these treatments. The second used “killed” mitochondrial treated with DNase to digest mtDNA. As expected, using “killed” mitochondria notably reduced the respiratory boost seen following transfer of live, respiring mitochondria. However, not until digesting mtDNA did we virtually eliminate any respiratory boost. Findings show living, respiring mitochondria offer the most oxidative support to host cells, but mtDNA within non-respiring mitochondria alone may confer respiratory benefit.

## 3. Discussion

Here, we show that newborn offspring exposed to PGDM have cardiac dysfunction and hypertrophy with inefficient ATP production and greater risk of stress-induced cell death. Mitochondrial transfer partially reverses the cellular phenotype in males but demonstrate a detrimental trend in female CM. These findings support our hypothesis that mitochondria play a central, pathogenic, and targetable role in developmentally programmed heart disease, and mitochondrial differences are not only exposure-related, but also sex-specific. This study sheds important light on mitochondria-mediated mechanisms of programmed cardiac disease that extend beyond impaired bioenergetics to include oxidative stress and faster cell death under metabolic stress.

This study builds upon foundational work in our lab which has characterized maternal, placental, and fetal outcomes in a rat model of STZ-induced LGDM, maternal HF diet, and the combination and repeatedly found that this combination increases perinatal mortality and causes cardiometabolic and mitochondrial dysfunction including impaired respiration and dynamism and oxidative damage in P1 offspring [8,9,10,12,35,36]. As in our LGDM model, cardiac function was impaired in PGDM-exposed offspring and especially in combination with maternal HF diet. However, unlike these previous studies, newborn offspring exposed to PGDM have less apparent bioenergetic dysfunction in vitro. We recently began using the Seahorse XFe which normalizes bioenergetics to the number of live cells at the end of the assay. Given that the MST exposes cells to FCCP and our in vitro FCCP Challenge increases cell death in PGDM-exposed CM, we surmise that bioenergetics data in this study are confounded by diabetes-mediated differences in cell viability. Indeed, not only do PGDM-exposed male CM consume more oxygen for ATP production on bioenergetics assays, but they have impaired ability to generate ATP, more oxidative damage, and faster mitochondria-mediated cell death following an FCCP Challenge. Impaired ATP generation in PGDM-exposed CM is supported by similar work by Petersen et al. who reported blunted ADP phosphorylation in skeletal muscle from insulin-resistant adults born to diabetic mothers [37]. It is well known that offspring of diabetic mothers develop responsive hyperinsulinemia in utero, which may “program” mitochondrial dysfunction including oxidative phosphorylation and viability. In our own studies, we have shown disruptions in cardiac insulin signaling pathways and relative insulin resistance in myocardium of offspring exposed to LGDM and maternal HF diet [11], so it is plausible that the hyperinsulinemia in our PGDM-exposed offspring is driving the mitochondrial effects shown here. 

Mitochondria are the biggest producers of cardiac ROS, and hydroxyl radicals damage mitochondrial proteins, mtDNA, and membrane lipids. The latter, termed lipid peroxidation, may impair mitochondrial functions including FA oxidation [38] and ATP production to potentially cause systolic dysfunction [10,39]. The significant oxidative damage found in PGDM-exposed CM may, therefore, be contributing to their functional cardiac deficits. Cardiolipin, a fatty acid found almost exclusively in the inner mitochondrial membrane, is highly susceptible to peroxidation [40] and is intimately involved with the release of cytochrome C, a precursor to mitochondria-mediated intrinsic apoptosis [41]. Although baseline differences in cytochrome C levels were not seen, our FCCP Challenge detected faster MMP loss and more cell death in PGDM-exposed CM subjected to metabolic stress, which is likely much more sensitive to detect a cellular vs. tissue difference compared to cytochrome C quantification. 

Diving deeper into mitochondria-mediated mechanisms of developmentally programmed heart disease, we augmented our bioenergetics assays and FCCP Challenge with mitochondrial transfer. After extensive validation, we found that mitochondrial transfer not only provides a respiratory boost but also improves viability in PGDM-exposed male CM. Interestingly, findings are sex-specific, and female recipients of mitochondrial transfer actually have a trend towards more apoptosis following stress. Findings support previous work from our lab [8,9] and others [42,43] that mitochondria have significant sexual dimorphism and contribute to sex-related differences in programmed cardiac disease. Moreover, this study suggests that mitochondrial transfer (transplantation), an emerging therapy, could have sex-divergent effects. Previous studies of mitochondrial transfer/transplantation in the context of treating IRI have identified similar benefits: transfer reduces oxidative stress-induced apoptosis per fewer TUNEL-positive cells [24], reduced caspase activity [20], and reduced myocardial necrosis by histologic staining [25] following IRI. Of note, the first study was performed in male rabbits whereas the latter two used female rabbits and pigs, respectively. All three used adult offspring, and there are certainly well-described metabolic differences in neonatal and adult CM [44,45]. Therefore, the discrepant effects of mitochondrial transfer found in females in our study (greater cell death) compared to IRI studies (reduced cell death) may be the result of age- or species-specific differences.

It is likely that the reduction in cell death in male PGDM-exposed CM results from multiple mechanisms. One mechanism demonstrated here is boosted respiratory capacity. Similarly, a 2015 study by Pacak et al. showed that mitochondrial transfer improves respiration of recipient HeLa cells [22]. A 2018 study by Kim et al. found that mitochondrial transfer to mesenchymal stem cells not only improves respiration but also enhances MMP and reduces ROS levels 48h after transfer [46]. A secondary but important finding from our study is that mtDNA may confer an important therapeutic benefit following mitochondrial transfer. While “killing” mitochondria offset much of the boost in cellular respiration, it was not until denaturing mtDNA that we negated respiratory effects to recipient cells altogether.

Despite mounting studies supporting efficacy [19,20,21,22,24,47], important challenges to mitochondrial transfer have been articulately expressed [48,49] and must be addressed. Pacak et al. and Kesner et al. have shown that mitochondrial transfer occurs through actin-mediated endocytosis and requires intact cellular heparin sulfate proteoglycans and outer mitochondrial membranes [22,50], but it remains a mystery as to how mitochondria escape Ca^2+^-induced permeability transition pore opening when traveling via vasculature to CM [48,49,51,52]. Considering that mitochondrial transplantation is currently under clinical trial in pediatric patients undergoing extracorporeal membrane oxygenation (ECMO) following myocardial IRI [27,28], it is imperative that studies confirm these mechanisms in vivo and solve the vasculature mystery. Nonetheless, in human clinical trials, transplants have significantly improved cardiac function, reduced the number of adverse cardiovascular events, and helped successfully wean pediatric patients off of ECMO to improve survival [28]. In addition to myocardial IRI, mitochondrial transfer/transplantation is being studied in the treatment of heart failure [53], diabetes-exacerbated IRI [47], acute limb ischemia [54], ischemic lung injury [55], and Alzheimer’s disease [56]. Here, we apply this emerging technique for mitochondrial dysfunction to developmentally programmed heart disease. Considering newfound sex-specific effects seen here, future studies should determine if sex of mitochondrial donors affects the outcomes of non-autologous mitochondrial transfer (rather than pooling sexes of donors as done here). Additionally, future studies should determine whether cardiomyopathy in offspring born to diabetic mothers improves with in vivo mitochondrial transplants. Finally, studies should further investigate whether developmentally programmed changes in mitochondria are inherited or acquired following exposure to in utero conditions. Reflecting metabolic memory mentioned above, we have previously found distinct, epigenetic signatures with maternal LGDM and HF diet that vary by solo and combination exposure [11,12]. The work of Miller and Orchard disputes the concept that short-term exposure to hyperglycemia alone causes disease attributed to metabolic memory; disease can instead be attributed to cumulative hyperglycemia exposure [57]. However, they also acknowledge that altered gene expression and oxidative stress, which are present in our study, result largely from other factors including epigenetics. That said, we expect PGDM conveys unique, fuel-mediated, inheritable changes to mitochondrial genes or mtDNA that effect long-term cardiac function despite euglycemic conditions after birth [18]. These efforts are underway in order to understand optimal timing and mechanisms for prevention.

## 4. Materials and Methods

### 4.1. Animal Model of PGDM and Maternal HF Diet

This study followed guidelines of the Animal Welfare Act and the NIH Guide for the Care and Use of Laboratory Animals in accordance with Sanford IACUC protocols 153-10-21B and 167-04-23B approved 28 November 2018 and 28 April 2020, respectively. Female, 8–10 week-old Sprague-Dawley rats (Envigo, Madison, WI, USA) received ad libitum control diet (CD) (TD2018, Envigo) or HF diet (TD95217, Envigo) for ≥28 days before breeding with normal, CD-fed males. At ≥5 days before breeding, dams received either citrate-buffer placebo or 65 mg/kg streptozotocin (STZ) to induce PGDM, thereafter treated with twice-daily sliding scale insulin to keep blood glucose levels from 200–400 mg/dL and prevent severe ketosis. One female received STZ but did not become diabetic (defined as blood glucose levels >200 mg/dL for ≥2 consecutive days) but received a second STZ injection before developing diabetes and breeding. 

Given a male predilection to developmentally programmed heart disease in humans and in our previous work, our first cohort examined only male P1 offspring from four distinct groups: controls (5 litters), PGDM-exposed (5), HF diet-exposed (6), and combination-exposed (5). After finding unexpected phenotypic differences in PGDM vs. our LGDM model, a second cohort was added to further characterize the effects of PGDM-mediated differences in both sexes. This was important because we previously showed female CM have better mitochondria quality control mechanisms [8,9]. This second cohort consisted of 5 control and 5 PGDM-exposed litters, but one diabetic dam miscarried all pups and was excluded. Each litter was matched with a timed-pregnant litter of pups (age P1 to P7; mean donor age P4) from a non-diabetic, control-fed dam, which served as donors of cardiac mitochondria for transfer. Following harvest and euthanasia of pups by cervical dislocation under isoflurane, dams were euthanized by cardiac removal under deep anesthesia to collect samples and count placentations, resorptions, and retained stillbirths for accurate measure of perinatal mortality.

### 4.2. Echocardiography

Cardiac structure and function were evaluated on the first cohort of P1 offspring using a Vevo 2100 Imaging System with MS700 MicroScan high-frequency transducer and Vevo LAB software (FUJIFILM VisualSonics Inc., Toronto, ON, Canada) [9,10,35].

### 4.3. Neonatal Ventricular Cardiomyocyte Isolation

Primary ventricular CM [8,9,10,44] were pooled from 3–5 male and female hearts/litter, counted by hemocytometry, and seeded to (1) FluoroDishes (ThermoFisher, Waltham, MA, USA) at 150,000 living CM/dish for confocal microscopy, (2) 24-well Seahorse XFe24 Microplates (Agilent, Santa Clara, CA, USA) at 80,000 living CM/well for extracellular flux analyses, (3) 35 mm plates at 1,000,000 CM/well for cytochrome C quantification, (4) 96-well black plates at 10,000 CM/well for ATP quantification, or (5) added directly to 1.5mL tubes at 300,000 CM/tube for malondialdehyde (MDA) quantification. CM for MDA quantification were assayed immediately, while seeded CM were allowed to adhere for ≥4 h. 

### 4.4. Mitochondrial Isolation, Transfer, and Treatment Validation

Immediately following euthanasia, donor mitochondria were isolated from normal newborn ventricular myocardium pooled from both sexes using Dounce homogenization and differential centrifugation as directed by Mitochondrial Isolation Kit for Tissue (Cat. #AB110168, Abcam, Cambridge, MA, USA). Approximately 8–10 rat hearts were pooled for each isolation, and samples were kept at 4^o^C throughout the isolation process. Final mitochondrial isolates were suspended in ice-cold respiration buffer [22] and quantified by DC protein assay (Bio-Rad, Hercules, CA, USA) and Cytation3 plate reader (BioTek, Winooski, VT, USA) as previously described [8].

Isolated mitochondria were aliquoted to be directly transferred to experimental CM or “killed” (snap frozen in liquid nitrogen to disrupt mitochondrial membranes and boiled at 95 °C for 5 min to denature all proteins) with or without treatment by 0.2% DNase I (to degrade mtDNA). As described below, 30 µg of mitochondria (live-respiring, “killed”, or “killed+DNase I-treated”) were added to each well containing 80,000 CM for Seahorse XFe24 bioenergetics assays, 60 µg of pHrodo Red-labeled mitochondria were added to FluoroDishes containing 150,000 CM for confocal imaging, and 60 µg of unstained mitochondria were added to similarly seeded FluoroDishes for FCCP Challenge. CM not receiving mitochondria received an equal volume of respiration buffer instead.

Before co-incubation with cultured CM, donor mitochondria used in imaging and validation studies were stained with 20 ng/mL pHrodo Red Succinimidyl Ester (Life Technologies, Grand Island, NY, USA), which fluoresces intensely when pH drops, such as during cellular internalization, as previously described [22]. Supernatant from the last wash was used as a negative control to show that no unbound pHrodo was transferred to CM (Figure S6A). Dilute HCl was used to drop the media pH (following co-incubation) to 4 as a positive control to identify pHrodo Red-labeled mitochondria outside of cells (Figure S6B). As additional validation of uptake and colocalization, we used *mito::mKate2* transgenic mice (Tg[CAG-mKate2]1Poche/J, stock #032188, JAX, Bar Harbor, ME, USA) as mitochondria donors. *Mito::mKate2* mice globally express the constitutively fluorescent protein mKate2, which is localized to the N-terminal cytochrome c oxidase subunit VIII, thus fluoresce without staining, removing confounding by dye leaching or variable pH (unlike pHrodo Red) [58]. Host CM used in confocal and validation studies were stained with 500 nM MitoTracker Green FM (for mitochondria) and 1 µM LysoTracker Blue DND-99 (for lysosomes; both from Invitrogen, Waltram, MA, USA) for 20 min followed by two washes in media. After baseline imaging of host (rat) CM, cells were co-incubated with *mito::mKate2* mouse mitochondria, “killed+DNase I-treated” *mito::mKate2* mitochondria, or media placebo (negative controls). After 1, 4, and 18 h of co-incubation, CM were washed twice to remove non-internalized mitochondria, imaged in 2D and 3D planes (Z-stack), trypsinized, and saved for qPCR quantification of mtDNA. Images at baseline, 4 h, and 18 h after co-incubation demonstrate that mitochondrial internalization was apparent at 4 h onwards (Figure S6C). 

Total DNA was isolated using QIAamp DNA Micro Kit (Cat. #56304, Qiagen, Germantown, MD, USA) per manufacturer’s instructions. Probe-primer sets specific to mouse and rat mtDNA sequences were selected (Table S2); mtDNA-encoded mouse cytochrome B (*mt-Cytb)* was chosen to quantify *mito::mKate2* donor mtDNA, and mtDNA-encoded rat *D-loop* was for host mtDNA [10]. After excluding species cross-reactivity (Figure S6D), qPCR was performed with 50 ng DNA/reaction using ABsolute Blue QPCR Mix on an ABI7500 thermocycler (ThermoFisher, Waltham, MA, USA). Data were analyzed as described by Quiros et al., 2017 [59] using reference gene *beta-2-microglobulin* and is shown in Figure S6E.

### 4.5. MDA, ATP, Cytochrome C, and Serum Metabolics Quantification

Lipid peroxidation was estimated using an MDA assay kit (Cat. #AB118970, Abcam, Cambridge, MA, USA) as previously described [10]. ATP levels with or without added ADP were quantified using Perkin Elmer ATPlite Luminescence Assay System (Cat. #50-904-9890, ThermoFisher, Waltham, MA, USA). After adding ADP or placebo (media) and ATP standards to isolated CM, the plate was mixed then incubated at room temperature for 10 min to allow CM time to phosphorylate ADP to ATP. Assay was then completed per manufacturer’s protocol. Alongside FCCP Challenge (below), CM for cytochrome C quantification were trypsinized, washed three times with 1× PBS, and permeabilized with 0.5% Triton X-100 before cryopreservation at −80 °C. Cell lysate cytochrome C levels were measured using R&D Systems Rat/Mouse Cytochrome C Quantikine ELISA Kit (Cat. #MCTC0, ThermoFisher, Waltham, MA, USA) per manufacturer’s instructions and normalized to protein content. Absorption and luminescence were measured using Cytation3 plate reader (BioTek, Winooski, VT, USA).

### 4.6. Plasma Analyses

Plasma fractions were stored at −80°C until analyses. Triglycerides, insulin, and C-peptide were quantified by Triglyceride Colorimetric Assay Kit (ThermoFisher, Waltham, MA, USA) and Rat Metabolic Hormone Panel (Cat# RMHMAG-84K-02, MilliporeSigma, Burlington, MA, USA) as previously described [9,10].

### 4.7. ATP Quantification for Respiratory Capacity and Immunoblotting for Purity of Isolated Mitochondria

To demonstrate retained respiratory capacity and ability to phosphorylate ADP, donor mitochondrial isolates were evaluated using ATPlite Luminescence Assay System as described above. Assay was performed immediately following mitochondrial quantification with the final mitochondrial pellet resuspended in respiration buffer made without ADP. Mitochondrial purity was determined using Western blotting for mitochondrial, nuclear, and cytoplasmic proteins as previously described [8,9]. Mitochondrial isolates were run alongside P1 whole heart homogenates with 40 µg protein per well. Blots were imaged using Luminata Forte HRP Chemiluminescence Substrate (ThermoFisher, Waltham, MA, USA) and ChemiDoc MP Imaging System (Bio-Rad, Hercules, CA, USA). Antibody information is listed in Table S3, and full-length blots are shown in Figure S7.

### 4.8. Live-cell Confocal Imaging for Quantification of Mitochondrial Uptake, Fusion, and Mitophagy

To quantify donor mitochondria internalization, fusion with host mitochondria, and mitophagy of donor and host mitochondria (per colocalization with lysosomes), stained CM were co-incubated with pHrodo Red-stained donor mitochondria. 3–10 cells/dish were imaged at baseline and after 1, 4, and 18 h co-incubation using an Eclipse A1R microscope and NIS-Elements software (Nikon Instruments Inc., Melville, NY, USA). Images were analyzed as previously described [9] using High-Content Screening Navigator (HCS) Colocalization.V4 protocol (Perkin Elmer, Waltham, MA, USA); non-internalized donor mitochondria were excluded. Baseline characteristics were evaluated using HCS Morphology.V4 protocol.

### 4.9. Bioenergetic Profiling

MST and GST were performed using a Seahorse XFe24 extracellular flux analyzer (Agilent, Santa Clara, CA, USA). To evaluate bioenergetic effects of mitochondrial transfer, CM from the same pool were similarly cultured with donor mitochondria or placebo respiration buffer. Both tests were started 4 h after mitochondrial transfer and assayed as previously detailed [9,10,44]. Proton efflux rate (PER) was calculated as described by Agilent Technologies [60] using a calculated Buffer Factor of 2.689533 mM/pH [61]. Media and injection strategies are detailed in Table S4.

### 4.10. FCCP Challenge to Measure Mitochondria-mediated Responses to Stress

Our FCCP Challenge has previously been described [9]. Here, following 18 h co-incubation with live, respiring mitochondria or placebo respiration buffer, CM were stained with 500 nM MitoTracker Green, 1 μM LysoTracker Blue, and 50 nM MitoTracker Red CMXRos (Invitrogen, Waltram, MA, USA), then incubated for 1h in MST media to simulate MST conditions. CM were imaged as previously described [9] except the final FCCP concentration was higher at 1.2 µM vs. 0.6 µM for adult CM as neonatal rat CM were more resistant to stress by respiratory uncoupling. Representative video-images are shown in Figure 5. Videos were analyzed using HCS Colocalization.V4 protocol to quantify MitoTracker Red intensity as an indicator of MMP at baseline and after FCCP uncoupling. Because neonatal CM were more resistant to FCCP-induced cell death than previously assessed adult CM [9] based on a dose response curve from 0.3–3.0 µM, rates of cell death could not be compared using retraction or pyknosis [9]. To quantify FCCP-stimulated cell death, following 25 min FCCP treatment CM were fixed in 4% PFA, permeabilized, and labeled with terminal deoxynucleotidyl transferase dUTP nick end labeling (TUNEL) and 4′,6-diamidino-2-phenylindole (DAPI) to distinguish apoptotic cells. TUNEL labeling was performed using Roche’s In Situ Cell Death Detection Kit, TMR Red (Cat#12156792910, MilliporeSigma, Burlington, MA, USA) by manufacturer’s instructions followed by DAPI labeling. Images were captured with an A1 TIRF Ti-Eclipse inverted confocal microscope (Nikon Instruments Inc., Melville, NY, USA) at 40× and analyzed using HCS CompartmentalAnalysis.V4 protocol. Apoptotic cells were defined as having total TUNEL fluorescent intensity >20,000 RFU.

## 5. Limitations of the Study

Similar to our studies of LGDM exposure [9,10], findings should be evaluated in other highly metabolic tissues such as the nervous system and liver before they can be applied outside the heart. While authors suspect that transfer of mtDNA benefits recipient cells, mtDNA has been found by others to have inflammatory properties that may contribute to development of cardiomyopathy when not properly contained within mitochondria or upon escape from lysosomes [62]. MtDNA may, therefore, be contributing to the increased apoptosis in recipient female CM, and future studies are needed to definitively rule out this possibility. Our studies did not investigate the effects of in vivo mitochondrial transplantation or efficacy differences that could result from same or divergent sex of donor and recipient cells. These studies are important and should be included in future work.

## 6. Conclusions

Ultimately, prenatal exposure to PGDM leads to impaired cardiac function that is worsened by maternal HF diet and associated with inefficient energy production, oxidative damage, and more mitochondria-mediated cell death following stress, especially in male offspring. Although the clinical phenotypes of human newborns exposed to PGDM and LGDM are similar, results from our two models show that underlying cardiometabolic and mitochondrial mechanisms are distinct, albeit potentially overlapping. Using mitochondrial transfer to isolate mitochondrial function, we found that donor mitochondria provide a respiratory boost to CM of all groups and reduce stress-induced apoptosis in males. While these findings support a pathogenic role of mitochondria in exposed offspring, future work should tease apart these sexually dimorphic differences, particularly our finding that transfer decreases apoptosis in males yet increases apoptosis in females.

## Figures and Tables

**Table 1:** Maternal and offspring characteristics.

	Parameter	N	Controls	PGDM-Exposed	Diet-Exposed	Combination -Exposed	Diabetes (*P* Value)	Diet (*P* Value)	Interaction (*P* Value)
**Dams**	Baseline wt., g	5–10	152 ± 3	170 ± 20	149 ± 5	174 ± 12	0.057	0.973	0.771
	Post-diet wt., g		217 ± 8	219 ± 11	224 ± 7	237 ± 7	0.370	0.150	0.505
	Glucose, mg/dL		94 ± 5	**^349 ± 21**	94 ± 2	**^413 ± 27**	N/A	N/A	**0.049**
	Ketones, mmol/L		0.8 ± 0.1	0.7 ± 0.1	0.6 ± 0.0	**^2.4 ± 0.3**	N/A	N/A	**<0.0001**
	Insulin need, units		0 ± 0	+85 ± 13	0 ± 0	**+115** **± 14**	**<0.0001**	0.120	0.120
	TG, mg/dL		70 ± 10	63 ± 7	*161 ± 25	*383 ± 115	0.075	**0.002**	0.060
	Litter size, pups		14 ± 1	12 ± 1	13 ± 2	13 ± 1	0.508	0.894	0.508
	Perinatal mortality rate, %	5–9	2.9 ± 1.9	12.0 ± 3.1	*15.4 ± 5.7	*19.3 ± 2.5	0.097	**0.014**	0.491
**Cohort 1 (Males)**	P1 weight, g	32–66	6.3 ± 0.1	**^6.9 ± 0.1**	6.4 ± 0.1	6.3 ± 0.2	N/A	N/A	**0.02**
	Glucose, mg/dL	24–37	91.4 ± 2.6	**+76.5 ± 2.1**	*94.6 ± 4.9	+*90.5 ± 2.5	**0.007**	**0.015**	0.124
	Insulin, pg/mL	5	756 ± 168	**+4505** **± 1113**	1062 ± 295	**+5201** **± 1709**	**0.002**	0.635	0.853
	C-peptide, pg/mL	5	2864 ± 616	**+6150** **± 690**	2660 ± 1149	**+3419** **± 768**	**0.027**	0.097	0.148
	^1^TG, mg/dL	5–6	108 ± 11	110 ± 21	129 ± 18	138 ± 24	0.776	0.219	0.844
	**Parameter**	**N**	**Controls** **Females Males**		**PGDM-Exposed** **Females Males**		**Diabetes** **(*P* Value)**	**Sex** **(*P* Value)**	**Interaction** **(*P* Value)**
**Cohort 2**	P1 weight, g	46–80	6.0 ± 0.9	6.3 ± 0.1	**+6.5 ± 0.1**	**+6.9 ± 0.1**	**<0.0001**	**0.012**	0.726
	Glucose, mg/dL	46–80	83.3 ± 2.1	88.3 ± 2.1	**+78.4 ± 3.0**	**+84.0 ± 2.4**	**0.018**	0.068	0.763
	^1^Insulin, pg/mL	4–5	1625 ± 358	1263 ± 376	**+4102 ± 1179**	**+3606** **± 788**	**0.002**	0.558	0.927
	^1^C-peptide, pg/mL	4–5	2723 ± 376	2467 ± 362	**+4277 ± 768**	**+4937 ± 879**	**0.003**	0.745	0.463
	^1^TG, mg/dL	5–6	138 ± 13	108 ± 11	94 ± 19	110 ± 21	0.212	0.682	0.179

^1^ Pooled serum samples from both cohorts. Perinatal mortality was calculated from both cohorts. TG, triglycerides; P1, perinatal day one; N/A, not applicable; PGDM, pregestational diabetes mellitus. Insulin need was from gestational day (GD)0 to GD22. Significant differences (*p* ≤ 0.05): ^+^ diabetes or * diet effect by 2-way ANOVA, ^ group effect remained significant by 1-way ANOVA and Dunnett post hoc test when significant interaction by 2-way ANOVA. Bold highlight significant differences. Sex-specific differences are indicated by *p*-value but are unmarked.

## Data Availability

Any data not included within the manuscript or Supplementary Information file are available from the corresponding author upon request.

## Supplementary Materials

The following are available online at https://www.mdpi.com/1422-0067/22/5/2382/s1.

## Abbreviations

LGDM: late-gestation diabetes mellitus; HF, high-fat; PGDM, pregestational diabetes mellitus; CM, cardiomyocyte; MMP, mitochondrial membrane potential; FCCP, carbonyl cyanide-4-phenylhydrazone; ROS, reactive oxygen species; IRI, ischemia-reperfusion injury; STZ, streptozotocin; HCS, High-Content Screening Navigator; MST, mitochondrial stress test; GST, glycolytic stress test; ECMO, extracorporeal membrane oxygenation.
