# Supplementary material for: Mitochondrial Transfer Improves Cardiomyocyte Bioenergetics and Viability in Male Rats Exposed to Pregestational Diabetes
Source: Int J Mol Sci. 2021 Feb 27;22(5):2382. doi: 10.3390/ijms22052382 (PMC7956857; doi:10.3390/ijms22052382)
Supplement: Supplementary file 1 [file ijms-22-02382-s001.pdf]

Supplementary Figures and Tables

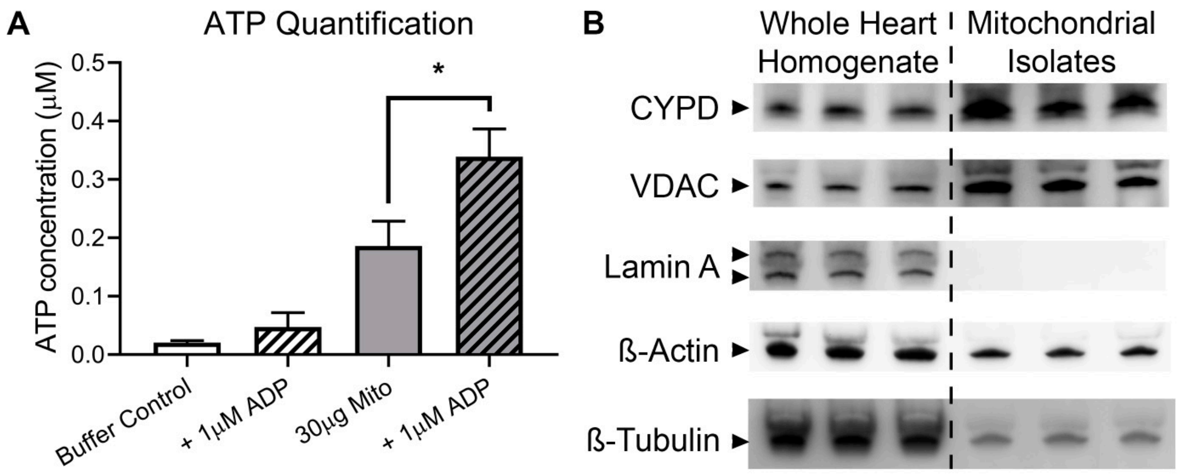

Figure S1. Isolated mitochondria retain respiratory capacity, are highly concentrated, and are relatively free of nuclear contamination. (A) Isolated mitochondria's ATP levels quantified with and without added ADP; *N* = 5 biological replicates. **(B)** Whole heart homogenate (left lanes) and mitochondrial isolates (right lanes) (separated by dashed line) were evaluated by immunoblotting for mitochondrial proteins cyclophilin D (CYPD) and voltage-dependent anion channel (VDAC), nuclear protein Lamin A, and cytoskeletal elements β-actin and β-tubulin. Data represent mean±SEM. *Significant difference ($P \leq 0.05$) by 1-way ANOVA.

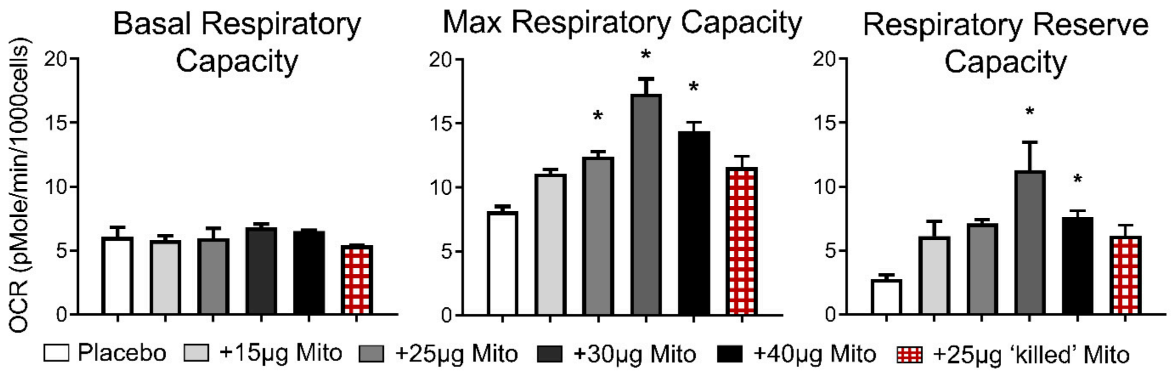

Figure S2. Donor mitochondria improve respiratory function of cardiomyocytes up to an optimal dose. Basal (left), maximal (center), and reserve respiratory capacity (right) were measured after 80,000 CM were treated with 15, 25, 30, or 40μg live, respiring mitochondria or 25μg 'killed' mitochondria. Placebo group received respiration buffer containing no mitochondria, and MST assay was run after four hours coincubation. Data represent mean±SEM. *N* = 2-3 per group. *$P < 0.05$ by 1-way ANOVA vs placebo control.

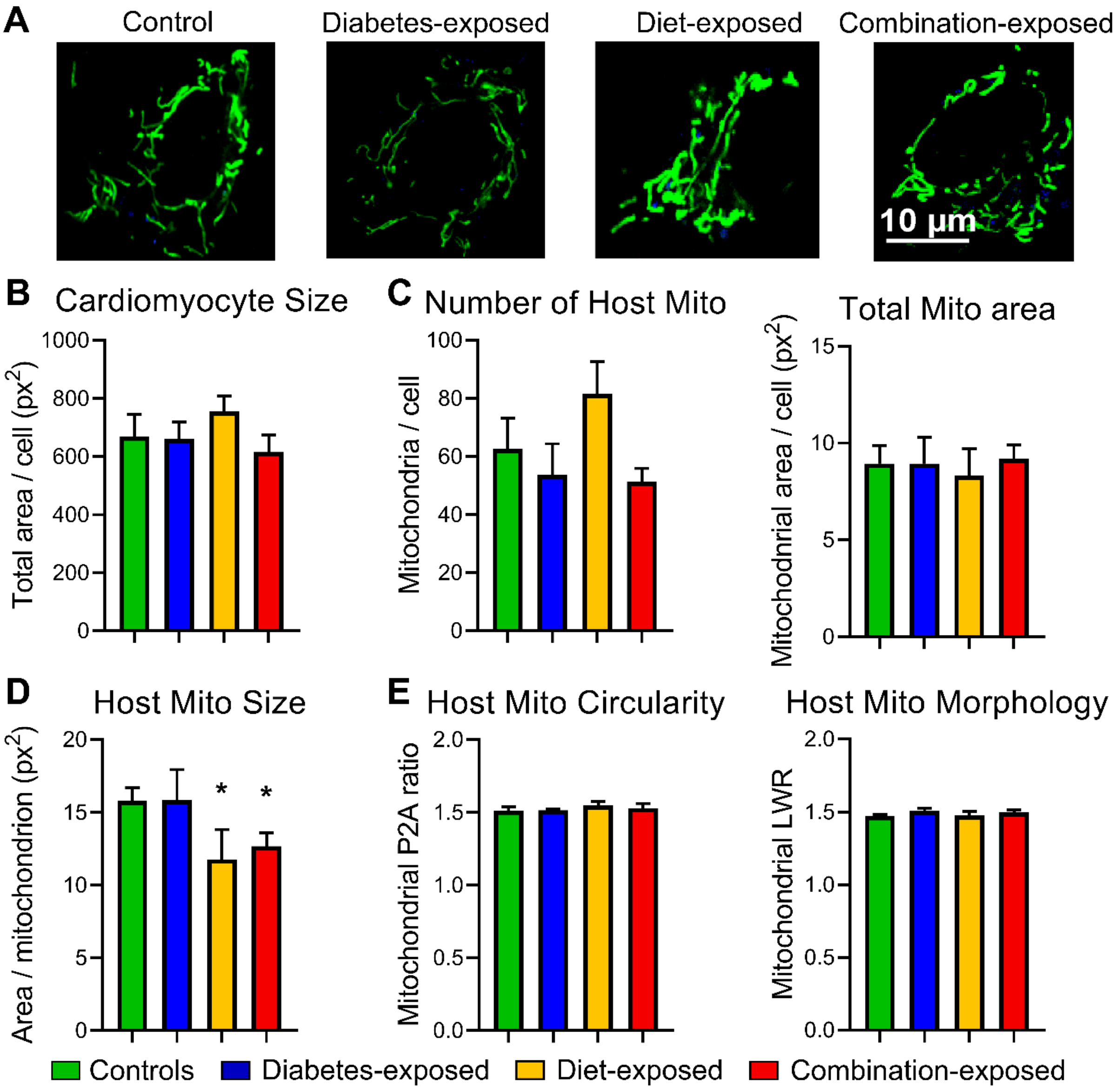

Figure S3. Control and exposed cardiomyocytes and mitochondria are morphometrically similar at baseline. (A) Representative images of cardiomyocytes from all groups stained with MitoTracker Green and LysoTracker Blue. Masked cells were evaluated using automated HCS software for cell size **(B),** number and size of host mitochondria **(C-D)**, and mitochondrial morphology **(E)**. Mitochondrial circularity ('roundness') and morphology were defined by P2A ratio (perimeter^2 / [4π*area]) and LWR (length:width ratio), respectively. Data represent mean±SEM. *N* = 5-6 biological replicates per group using 3-10 randomly selected cells per replicate. Significant differences ($P \leq 0.05$): *diet-specific effect by 2-way ANOVA.

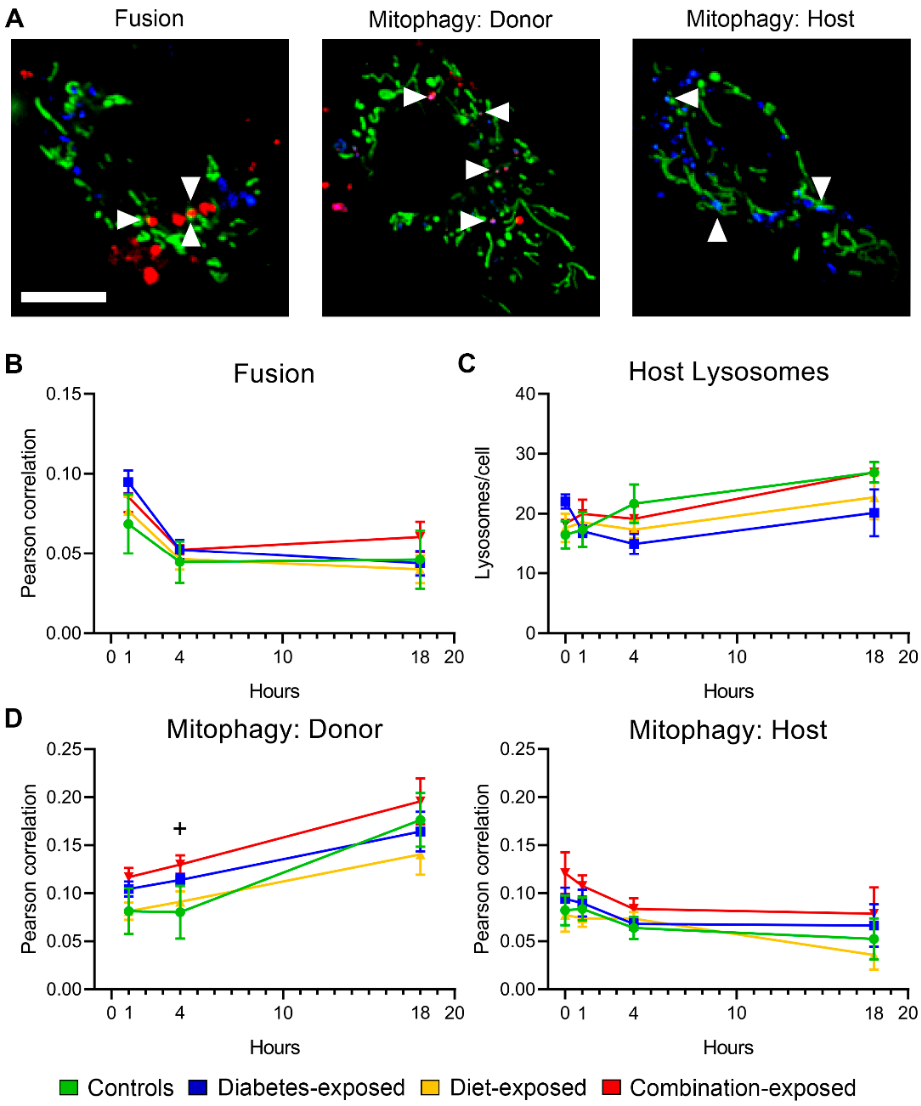

Figure S4. Mitochondrial transplantation leads to mitochondrial fusion and decreased mitophagy of host mitochondria. (A) Representative confocal live-cell images of colocalized host and donor mitochondria indicating fusion (first panel), donor mitochondria (red) and host lysosomes (blue) and host mitochondria (green) and lysosomes (blue) representing mitophagy. Graphs illustrate **(B)** co-localization coefficient of donor and host mitochondria, a marker of mitochondrial fusion, **(C)** total numbers of host lysosomes and **(D)** lysosomes colocalized with donor (left) and host (right) mitochondria, a marker of mitophagy, at baseline, 1, 4 and 18 h post mitochondrial transplants. Linear slopes represent fusion, lysosome production and mitophagy rates over time. Data represent mean±SEM. *N* = 5-6 males/group. $^{+}$Significant diabetes effect ($P \leq 0.05$) by 2-way ANOVA. Significant differences within groups at different time points are indicated in text above. Scale, 10μm.

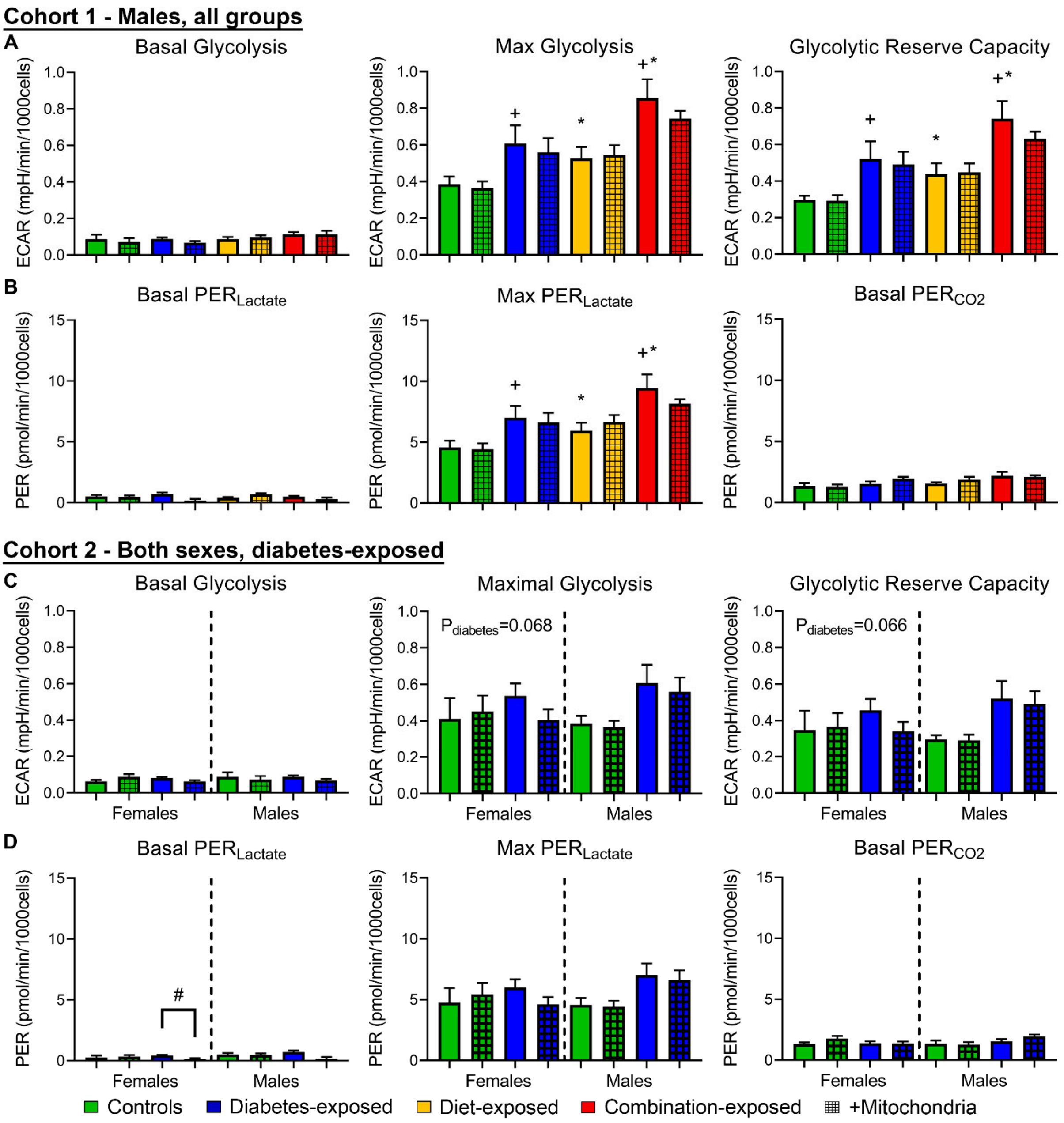

Figure S5. Male cardiomyocytes exposed to pregestational diabetes and maternal high-fat diet are more glycolytic than controls. Glucose-stimulated basal, monensin + rotenone-antimycin A-stimulated maximal, and reserve glycolytic capacities in all four groups of male cardiomyocytes (CM) **(A)** and control and diabetes-exposed CM of both sexes **(C)**. Proton efflux rate (PER) differentiates acidification due to lactate (anaerobic glycolysis) vs CO_2 (aerobic respiration) in both cohorts **(B, D)**. *N* = 4-6 per sex per group. Data represent mean±SEM. $P \leq 0.05$: $^+$diabetes or *diet effect by 2-way ANOVA, $^\#$mitochondrial effect by 1-way ANOVA.

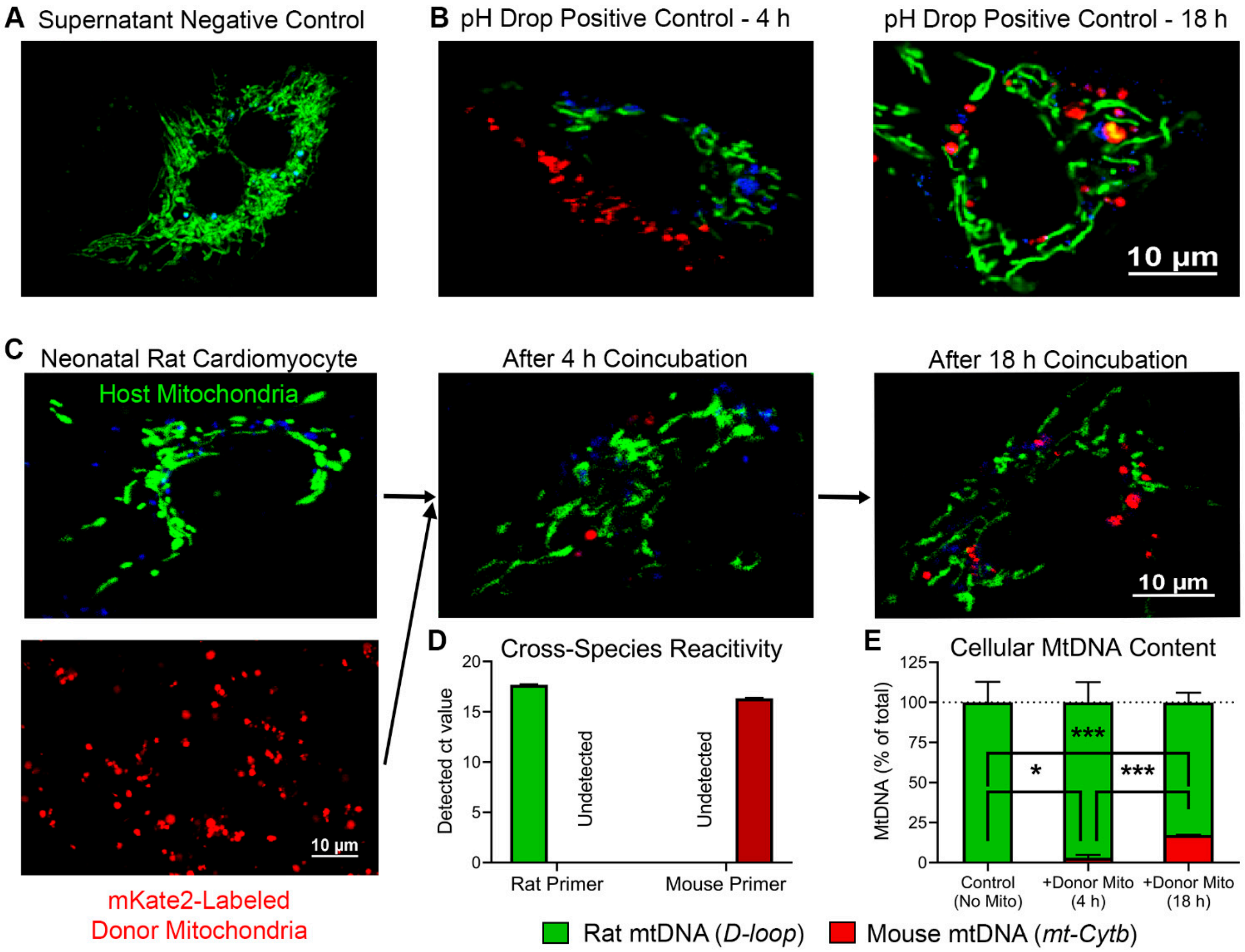

Figure S6. Validation of mitochondrial transplantation using pHrodo Red-labeled rat mitochondria and mKate2-labeled mouse mitochondria. (A-B) Representative images of CM stained with MitoTracker Green and LysoTracker Blue and incubated with either the fourth wash supernatant (negative control) from pHrodo-staining **(A)** or pHrodo Red-labeled donor mitochondria **(B)**. As pHrodo Red is a pH-dependent stain, media pH was dropped to 4 after 4 h (middle) and 18 h (right) coincubation to show presence of donor mitochondria both around and inside CM. **(C)** Representative images of CM (top left), isolated myocardial mitochondria from *mito::mKate2* mouse (bottom left), and coincubation of the two after four (top middle) and 18 hours (top right). Middle and right images were taken after washing off non-internalized mkate2 mitochondria but before trypsinization. **(D)** Real-time quantitative PCR (qPCR) of host rat cardiomyocyte and donor mouse DNA show that species-specific mitochondrial (mt)DNA-targeted probe/primer sets do not cross-react. **(E)** qPCR for mtDNA at baseline (placebo injection) and after 4 and 18 h of coincubation with mkate2 mouse mitochondria shows relative levels of internalized donor mitochondria relative to total mtDNA (set at 100%). Data represent mean±SEM. *$P \leq 0.05$ by 1-way ANOVA. $N = 3$ experimental replicates.

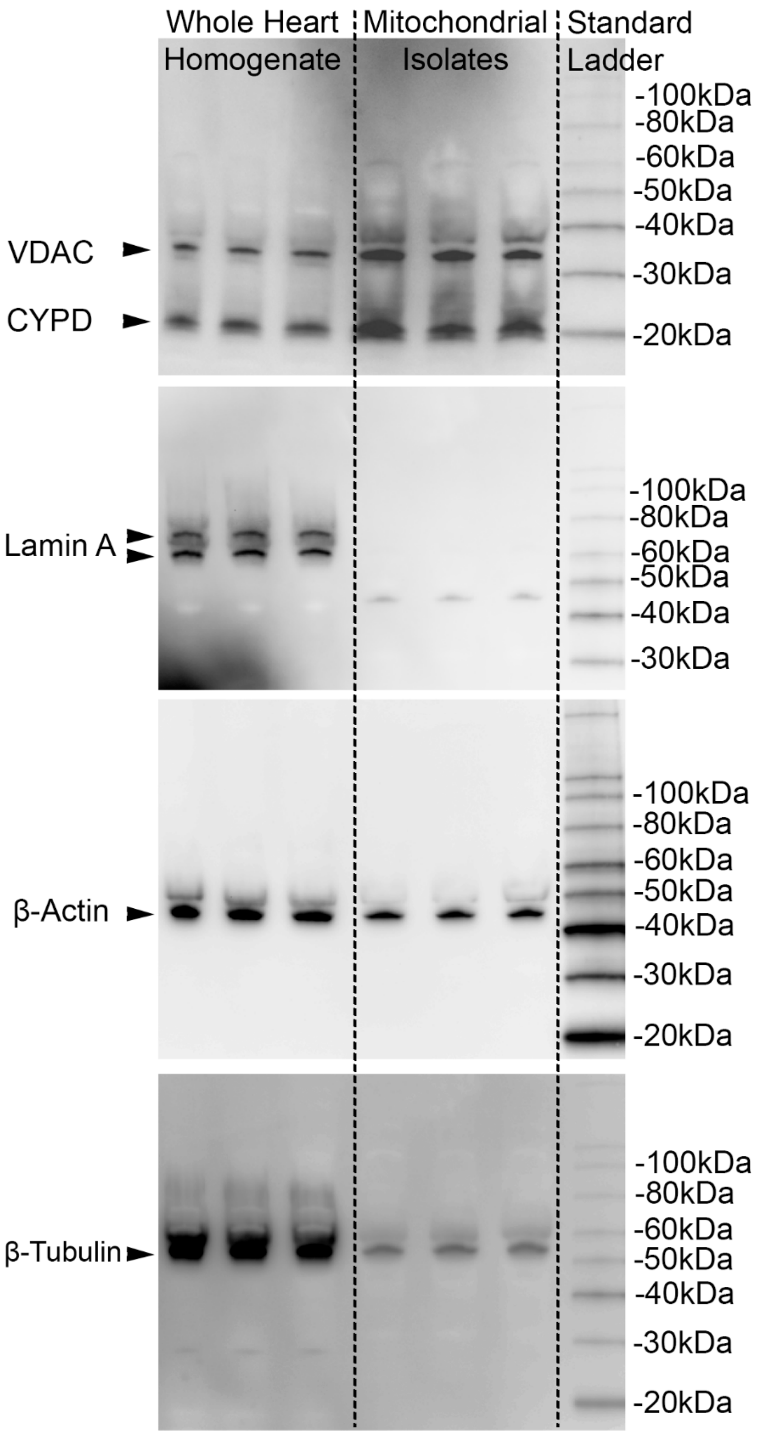

Figure S7. Full-length, unaltered immunoblots from Figure S1. Bands of interest and molecular weight ladders are marked on the left and right sides of blots, respectively.

Table S1. Offspring morphometric and echocardiographic results.

	Parameter	*N*	Controls	PGDM-exposed	Diet-exposed	Combination-exposed	Diabetes (*P value*)	Diet (*P value*)	Interaction (*P value*)
Cohort 1 (male offspring)	Heart:body wt ratio (x10^{-3})	37-61	7.3±0.1	**+7.5±0.1**	+7.1±0.1	**+7.5±0.1**	**0.008**	0.3.7	0.536
	EF, %		76.1±1.5	**+69.0±1.6**	73.6±2.0	**+66.1±1.4**	**<0.0001**	0.125	0.903
	FS, %		43.6±1.4	**+37.6±1.3**	41.9±1.7	**+35.2±1.0**	**<0.0001**	0.148	0.819
	E:A ratio		0.71±0.03	0.73±0.01	0.72±0.02	0.65±0.03	0.364	0.146	0.073
	HR, bpm		254±5	262±6	258±8	260±7	0.512	0.967	0.693
	SV, µL		24.1±0.9	24.6±1.1	***20.9±0.8**	***20.4±0.8**	0.997	**<0.001**	0.579
	CO, mL/min		6.1±0.2	6.4±0.2	***5.4±0.3**	***5.3±0.3**	0.754	**0.002**	0.541
	LV mass, mg		44.8±1.5	49.1±4.3	41.3±1.6	46.2±2.8	0.083	0.223	0.909
	IVSd, mm		0.60±0.03	0.57±0.02	0.60±0.02	0.57±0.02	0.160	0.967	0.943
	LV diastolic diameter, mm		2.88±0.04	**+3.02±0.04**	***2.76±0.05**	**+*2.84±0.04**	**0.022**	**0.003**	0.596
	LV diastolic volume, µL		31.8±1.2	**+35.6±1.2**	***28.8±1.3**	**+*30.8±1.0**	**0.021**	**0.003**	0.483
	PAAT:RVET ratio	12-19	0.22±0.02	0.18±0.01	0.20±0.01	0.21±0.01	0.534	0.709	0.091
	**Parameter**	***N***	**Controls Females**	**Males**	**PGDM-exposed Females**	**Males**	**Diabetes (*P value*)**	**Sex (*P value*)**	**Interaction (*P value*)**
Cohort 2 (both sexes)	Heart:body wt ratio (x10^{-3})	44-61	7.5±0.1	7.3±0.1	7.6±0.1	7.6±0.1	0.372	0.236	0.506
	EF, %		75.0±0.8	75.4±3.1	76.9±1.1	75.2±0.8	0.559	0.646	.0496
	FS, %		42.4±0.7	43.6±2.9	44.4±1.0	42.6±0.7	0.698	0.846	0.290
	E:A ratio		0.73±0.02	0.79±0.02	0.79±0.06	0.79±0.02	0.4772	0.540	0.488
	HR, bpm		283±7	262±8	291±9	287±9	0.062	0.146	0.312
	SV, µL		23.5±1.4	23.6±1.8	**+27.7±1.1**	**+25.3±0.9**	**0.0374**	0.424	0.3835
	CO, mL/min		6.7±0.5	6.2±0.6	**+8.1±0.4**	**+7.2±0.3**	**0.018**	0.185	0.736
	LV mass, mg		30.5±1.2	34.0±2.1	**+42.7±4.0**	**+40.7±2.4**	**0.002**	0.780	0.350
	IVSd, mm		0.40±0.01	0.51±0.04	**+0.57±0.05**	**+0.54±0.03**	**0.022**	0.327	0.075
	LV diastolic diameter, mm		2.85±0.07	2.86±0.08	**+3.02±0.05**	**+2.95±0.04**	**0.042**	0.593	0.533
	LV diastolic volume, µL		31.4±2.0	31.5±2.3	36.0±1.4	33.8±1.2	0.062	0.564	0.524
	PAAT:RVET ratio	10-15	0.29±0.01	0.27±0.03	0.29±0.02	0.25±0.02	0.678	0.148	0.575

EF, ejection fraction; FS, fractional shortening; E:A ratio, ratio of ventricular filling velocities in early to late diastole; HF, heart rate; SV, stroke volume; CO, cardiac output; LV, left ventricle; IVSd, interventricular septum thickness during diastole; PAAT:RVET ratio, ratio of pulmonary artery acceleration time to right ventricular ejection time. Significant differences ($P \leq 0.05$): $^{+}$diabetes or *diet effect by 2-way ANOVA.

Table S2. Probe-primer sets used in *mito::mKate2* mouse validation experiments.

Gene	Species	Source	Identifier, Assay ID, and/or Sequence
B2m	Rat, Mouse	Integrated DNA Technologies	NM_012512.2; Rn03928990_g1
mtDNA D-loop	Rat	Integrated DNA Technologies	Custom-designed assay;<br>Probe: /56-FAM/TTGGTTCAT /ZEN/CGTCCATACGTTCCCCTTA/3IABkFQ/<br>Primer 1: GATTAGACCCGTTACCATCGAGAT<br>Primer 2: GGTTCTTACTTCAGGGCCATCA
Rlp4	Mouse	Bio-Rad	ENSMUST00000034966; qMmuCEP0034839
mt-CytB	Mouse	Bio-Rad	ENSMUST00000082421; qMmuCEP0033357

Table S3. Antibodies used in Western blotting analyses.

Antibody	Source	Identifiers
Anti-VDAC1/Porin, diluted 1:1000	Abcam	Cat#Ab14734; RRID:AB_443084
Anti-CYPD, diluted 1:1000	Abcam	Cat#Ab110324; RRID:AB_10864110
Anti-LaminA/C, diluted 1:1000	Cell Signaling Technology	Cat#2032S; RRID:AB_2136278
Anti-β-Actin (HRP-conj.), diluted 1:1000	Cell Signaling Technology	Cat#5125S; RRID:AB_1903890
Anti-β-Tubulin (HRP-conj), diluted 1:1000	Cell Signaling Technology	Cat#5346; RRID:AB_1950376
Goat anti-rabbit IgG-HRP, diluted 1:5000	SouthernBiotech	Cat#4030-05; RRID:AB_2687483
Goat anti-mouse IgG(H+L) human ads-HRP, diluted 1:5000	SouthernBiotech	Cat#1031-05; RRID:AB_2794307

Table S4. Media details and injection strategies for bioenergetic profiling assays.

Assay	Media	Port A	Port B	Port C	Port D
**Mitochondrial Stress Test**	XF DMEM Media (Agilent)<br>4mM L-glutamine<br>10mM D-(+)-glucose<br>1mM pyruvate	2μM oligomycin	0.3μM FCCP	2μM rotenone<br>4μM antimycin A	2μM Hoechst 33342
**Glucose Stress Test**	XF DMEM Media (Agilent)	10mM glucose	20μM monensin	2μM rotenone<br>4μM antimycin A	3.8mM 2-deoxyglucose<br>2μM Hoechst 33342

Port injections represent final well concentrations of each reagent.
